# Multifunctional and Environmentally Friendly TiO_2_–SiO_2_ Mesoporous Materials for Sustainable Green Buildings

**DOI:** 10.3390/molecules24234226

**Published:** 2019-11-20

**Authors:** Elena Ghedini, Federica Menegazzo, Maela Manzoli, Alessandro Di Michele, Debora Puglia, Michela Signoretto

**Affiliations:** 1 CATMAT Lab, Department of Molecular Sciences and Nanosystems, Ca’ Foscari University Venice and Consortium INSTM, RU of Venice, Via Torino 155, 30172 Venezia, Italy; elena.ghedini@unive.it (E.G.); federica.menegazzo@unive.it (F.M.); 2 Department of Drug Science and Technology & NIS—Centre for Nanostructured Interfaces and Surfaces, University of Turin, Via P. Giuria 7, 10125 Turin, Italy; maela.manzoli@unito.it; 3 Department of Physics and Geology, University of Perugia, Via Pascoli, 06123 Perugia, Italy; alessandro.dimichele@unipg.it; 4 Civil and Environmental Engineering Department, University of Perugia, UdR INSTM, Strada di Pentima 4, 05100 Terni, Italy; debora.puglia@unipg.it

**Keywords:** TiO_2_, silica, VOC, photocatalysis, surface area, anatase, green building, insulating

## Abstract

This work deals with the formulation of environmentally friendly, cheap, and readily-available materials for green building applications, providing the function of air purificator by improving the safety and the comfort of an indoor environment. High surface area TiO_2_–SiO_2_ samples, prepared by a simple, cost effective, and scalable synthetic approach, proved to be effective in maximizing the properties of each component, i.e., the photocatalytic properties of titania and the high surface area of silica. TiO_2_ was introduced onto an ordered mesoporous silica Santa Barbara Amorphous-15 (SBA-15), that is featured by interesting insulating features, by using an incipient wetness impregnation method. The photocatalytic activity was evaluated in gas phase oxidation of ethylbenzene, which was selected as model volatile organic compound (VOC) molecule. The morphological, textural and structural features along with the electronic properties, the hydrophilicity and heat capacity of the materials were investigated in depth by scanning electron microscopy, powder X-ray diffraction, N2 physisorption, diffuse reflectance UV-Vis, FT-IR spectroscopies, and modulated DSC (MDSC) dynamic scan. Outstanding performances in the ethylbenzene abatement results are promising for further application in the green building sector.

## 1. Introduction

Buildings are responsible for a huge share of energy, electricity, water, and materials consumption worldwide: 20% of global energy is consumed by the building industry and this value is expected to grow annually up to about 30% in 2040 [1,2,3]. Moreover, buildings account for 18% [4] of global emissions every day, that corresponds to 9 billion tons of CO_2_ emitted annually. Unless new technologies in construction are adopted during this time of rapid growth, emissions could double by 2050, according to the United Nations Environment Program [5]. Green building practices are aimed at reducing the environmental impact of building and maximizing, at the same time, comfort and safety of the living spaces. In particular, the new building trends are focused on innovative technologies and materials to meet more recent and more demanding efficiency and environmental regulations [6,7]. The indoor environmental quality (IEQ) design and construction guidelines are centered on four pivotal issues: (i) indoor air quality (IAQ), (ii) thermal quality, (iii) lighting quality, and (iv) energy efficiency. In particular, IAQ relies on reducing the amount of volatile organic compounds (VOCs) and other air impurities, such as microbial contaminants.

The production and use of most building materials and cleaning/maintenance products emit gaseous, some of them toxic, compounds such as many VOCs, NOx, which have a detrimental impact on occupants’ health, comfort, and wellbeing [8]. Therefore, the choice of construction materials and interior finish products with zero or low VOC emissions during their use might enhance IAQ. Another successful strategy could be the use of interior cladding that can promote the pollutants abatement by improving the in-door safety. To achieve this ambitious goal, the paradigms of sustainability and cheapness must be irrevocably satisfied. Indeed, environmentally friendly and cost-effective materials and technologies are required to abate their impact in terms of non-renewable resources employed and energy consumption [9]. Consequently, the alternative use of technologically advanced yet affordable materials able to degrade pollutants by a photocatalytic process is a promising approach. In this frame, material formulation must be optimized in order to assure good photocatalytic performances and at the same time to guarantee the preservation, and when possible, to ameliorate the energy efficiency of the building.

Among the most known photocatalysts, TiO_2_ is characterized by high photocatalytic activity in oxidation reactions that, along with its non-toxicity, good availability, low cost, and photochemical stability in reaction conditions, is the most-employed photocatalyst in the abatement of atmospheric pollutants [10,11]. It is now well established that TiO_2_ photocatalytic activity strongly depends on its physical properties, such as crystal structure, crystallite size, and surface hydroxyl groups. Indeed, a good photocatalyst for VOCs abatement should be featured by high specific surface area in order to favor the adsorption of pollutants, which proved to be the most crucial step of the whole process [12]. Moreover, high surface area and elevated porosity are among the requirements to achieve building additive with good insulating features [13].

Several efforts have been devoted at optimizing an effective synthetic approach in order to obtain TiO2 materials with high surface area. In recent years, the feasibility of synthesizing ordered mesoporous titania has prompted a great interest in the field of photocatalytic applications, because of the combination of high surface area and narrow distribution of the pore diameters, possibly with a well-defined crystalline structure. Through the modification of the sol-gel synthesis, Antonelli and Ying [14] prepared a mesoporous material based on amorphous titanium dioxide by controlling the hydrolysis of Ti(OiPr)_4_ using acetylacetone as a chelating agent in the presence of tetradecylphosphate, that led to the presence of phosphorus traces in the final photocatalyst depressing the activity greatly [15]. Then, several synthetic approaches have been attempted by using several cationic structure directing agents or surfactants [16,17], neutral surfactants [18], and inorganic salts as precursors of the metal in non-aqueous solutions [19,20], but none of these have produced materials with short-range order. In 2010, Kao et al. [21] published a study on the effect of the aging conditions on the synthesis of ordered mesoporous TiO_2_ with high surface area, whereas Hung et al. [22] reported the influence of the surfactant concentration on the final structure. Moreover, the development of a methodology for the synthesis of TiO_2_ using structure directing agents would require time consuming optimization of the procedure, resulting in high costs of the final material. The introduction of TiO_2_ nanoparticles on ordered mesoporous silica, like Santa Barbara Amorphous-15 (SBA-15) [23,24], or MCM-41 [25], whose synthesis authors have experience with [26], represents an alternative, simpler, and less expensive approach. Ordered mesoporous silica materials [27] would be a good and cheaper alternative to very expensive nanostructured porous materials such as silica aerogels [28] that are the most efficient materials for thermal insulation (k(silica aerogel) = 10 mW∙m^−1^∙K^−1^, k(fumed silica) = 20 mW∙m^−1^∙K^−1^). These materials have a large specific surface area (>1000 m^2^∙g^−1^) with a high pore volume (>1 cm^3^∙g^−1^). Studies on thermal conductivity of thin-films made up by ordered mesoporous solids reported that cubic and hexagonal ordered mesoporous films have thermal conductivity ranging between 0.2–0.3 W∙m^−1^∙K^−1^ [29]. A thermal conductivity as low as 0.032 W∙K^−1^∙m^−1^ was also measured for mesoporous silica nanoparticles thermally treated at 550 °C. Moreover, much lower thermal conductivity is measured for powders compared to thin films, since in the former there is an important textural porosity that contributes to lower the thermal conductivity. Finally, these materials proved to be fire- and draught-proof, differing from generally-used insulators in green building, like wool, cellulose, and straw, which require a time-consuming treatment to be used [30].

In a previous work we investigated TiO_2_/MCM-41 systems in the photocatalytic abatement of NO_X_ [12]. Generally, the synthesis of MCM-41, as reported by Wang et al. [31], involves the use of a “ionic” surfactant, usually hexadecyltrimethylammonium bromide (CTA-Br) as a structure directing agent. The range of surfactants available for such use is quite wide. In this context, the poly(ethylene oxide)n-poly(propylene oxide)x-poly(ethylene oxide)y, (PEO)x(PPO)y(PEO)x (PLURONIC P123) plays a major role, being used in the synthesis of SBA-15.

It is worth noting that the non-ionic surfactants have the advantage of being non-toxic, biodegradable, and cheaper compared to the ionic surfactants commonly used. Therefore, SBA-15 was chosen as silica material for supporting TiO_2_ [32]. Santa Barbara Amorphous-15 (SBA-15) is a highly stable mesoporous silica sieve developed by researchers at the University of California at Santa Barbara. It gets its high hydrothermal and mechanical stability from a framework of uniform hexagonal pores that feature a narrow pore-size distribution and a tunable pore diameter of 5 nm to 15 nm, but, most significantly, from its relatively thick walls, which range between 3.1 nm and 6.4 nm. SBA mesoporous silica 15 has a high internal surface area, which lends itself to various applications, including environmental adsorption and separation, advanced optics, and catalysts. [24]. In most cases, the synthetic procedure, for the preparation of TiO2/SBA-15 composites, was based on sol-gel or co-precipitation methods and the porosity and surface area of the final materials were not satisfactory (generally between 200 and 500 m^2^/g). Therefore, the incipient wetness impregnation of TiO_2_ on SBA-15 could be a simple and effective method to obtain composites with high surface area and porosity and, at the same time, to improve the photoactivity for pollutant degradation.

The aim of the present paper is the formulation of an environmentally friendly, cheap and multi-functional material for application in the building sector. High surface area silica–titania systems were produced by a cost-effective approach and their properties were investigated to evaluate their effectiveness in abating pollutants and acting as insulating materials as well.

## 2. Discussion

### 2.1. Preliminary Photocatalytic Studies and Characterization of the Commercial TiO_2_ Samples

**Figure 1:**
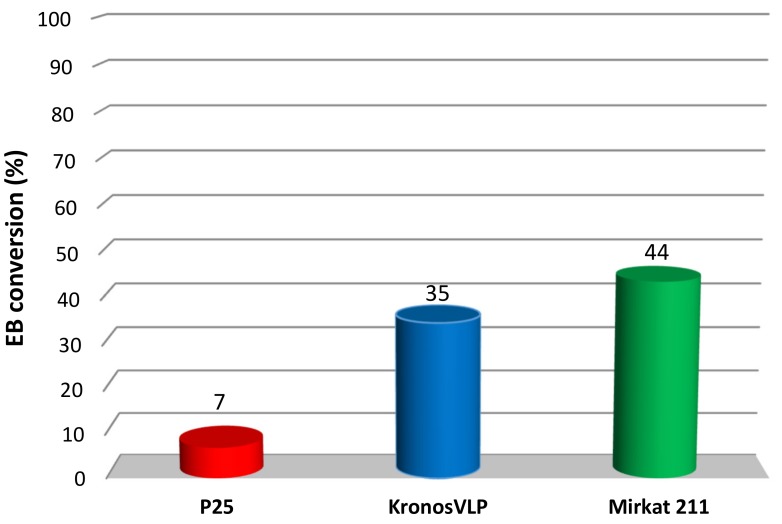
Ethylbenzene (EB) photocatalytic degradation obtained for the three TiO_2_ commercial catalysts.

In order to establish a correlation between the photocatalyst features and the photocatalytic activity, a preliminary screening was performed on the commercial samples by investigating the catalytic performance in the photo degradation of ethylbenzene (EB) and the results are shown in Figure 1.

Very different EB conversions were observed for the three materials. In particular, P25 Degussa, which is usually regarded as the reference commercial photocatalyst, showed the lowest conversion (7%). On the contrary, Kronos VLP and Mirkat 211 reached 35% and 44% conversion, respectively. As already discussed, the activity of TiO_2_ is related to various factors. Among these, the crystalline phase and the surface area are determinant [12]. Therefore, N_2_ physisorption, SEM and XRD analyses were performed on the commercial samples to investigate such features.

It is worth noting that the reaction here considered (EB photo-oxidation) occurs through a heterogeneous catalytic process that strongly depends on the surface area of the catalyst. The photocatalytic degradation of pollutants is based on two steps and the first one foresees the absorption of VOCs on the semiconductor surface (see Paragraph 2.2). As reported in Table 1, the BET surface area values and the pore volume obtained for Kronos VLP and Mirkat 211 commercial materials are very similar (above 200 m^2^/g), whilst P25 has a specific surface area of 52 m^2^/g and a significantly lower pore volume.

However, the sample characterized by the highest surface area did not provide the greatest photoactivity, indicating that this parameter, though being extremely important, is not sufficient to provide satisfactory activity. First of all, according to the SEM images reported in sections a and b of Figure S1, both Kronos VLP and Mirkat 211 samples are made of non-homogeneous particles with size of several tens of nanometers, whilst large agglomerates of particles that are characterized by jagged edges were observed in the case of P25 (section c of the same Figure). This morphology is consistent with the presence of a certain fraction of rutile crystal phase (see XRD patterns) that is generally characterized by granules larger than those belonging to anatase one [33].

**Figure 2:**
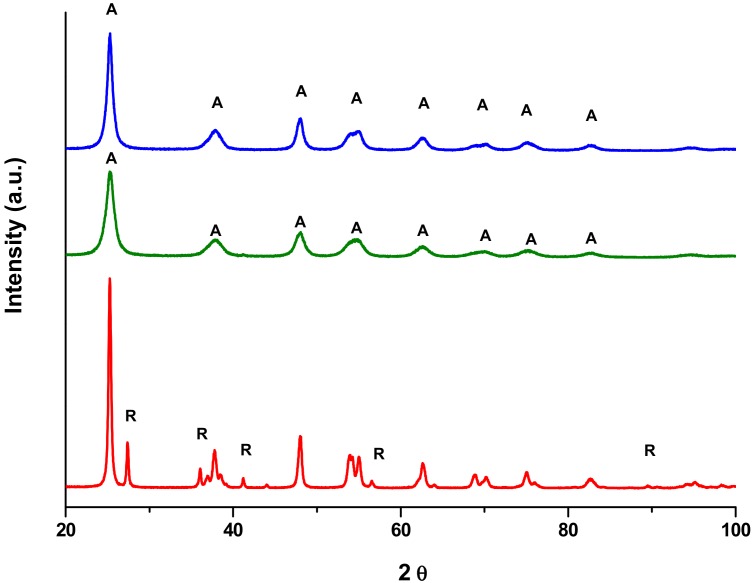
XRD patterns of P25 (red line), Kronos VLP (blue line) and Mirkat 211 (green line) commercial samples.

The presence of the anatase crystal phase in all the commercial catalysts was confirmed by XRD, as shown in Figure 2. Moreover, weak peaks ascribable to the rutile phase were also observed in the pattern of P25 (red line), in agreement with SEM findings. The peaks related to Kronos VLP (blue) and Mirkat 211 (green line) commercial samples are not well resolved and this feature indicates the presence of very small titania nanocrystals (according to the estimated particle sizes reported in Table 1).

**Figure 3:**
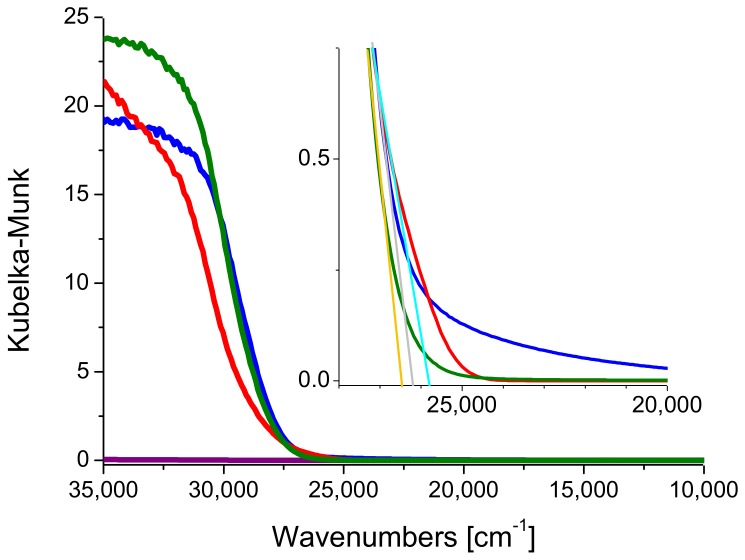
DRUV-Vis spectra of P25 (red line), Kronos VLP (blue line) and Mirkat 211 (green line). The spectrum of Santa Barbara Amorphous-15 (SBA-15) (purple line) is reported for the sake of comparison. Inset: zoom on the band-gap.

The electronic properties of the commercial materials were investigated by DRUV-Vis spectroscopy and the measurements were carried out in air and at room temperature. The results are shown in Figure 3, in which the spectrum of bare SBA-15 is also reported for comparison purposes.

The band-gap values of Kronos VLP (blue line) and Mirkat 211 (green line) were graphically extrapolated and it was found that close to the reference value reported for anatase (3.2 eV) [34]: A band gap of 3.21 eV and 3.25 eV was obtained respectively (see insert Figure 3). On the contrary, P25 showed a band gap of 3.15 eV, possibly due to the presence of rutile, whose band gap is lower than anatase (3.0 eV vs. 3.2 eV).

Basing on the overall findings, performing catalysts must fulfill (i) high surface area to improve EB adsorption and (ii) high crystallinity (anatase phase) to increase the photodegradation activity. However, the surface area of the investigated samples was not high enough also to provide suitable insulation properties [35]. For this reason, silica–titania materials represent a promising option to further improve the surface area and, as a consequence, both EB adsorption and insulation properties.

### 2.2. High Surface Area TiO_2_/SBA-15 Materials

**Figure 4:**
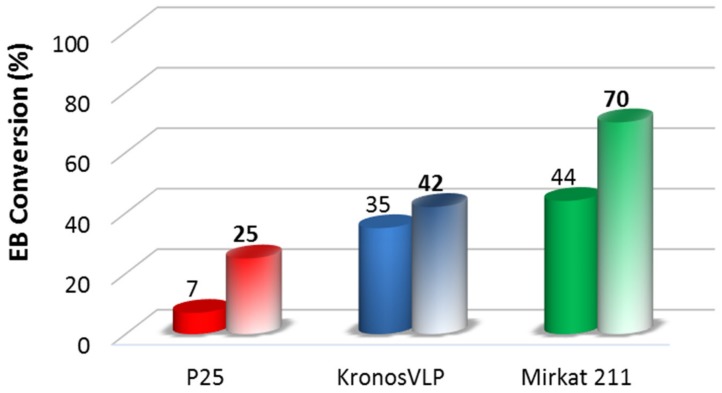
EB photocatalytic degradation obtained for the TiO_2_ commercial catalysts (full colors) compared to that obtained for the TiO_2_/SBA-15 materials (soft colors).

The photocatalytic activity of the composite systems was investigated, and the results are shown in Figure 4. The combination of titania and silica positively influenced the photocatalysis, since all TiO_2_/SBA-15 materials show conversion values higher than those related to the bare commercial ones. Such synergic effect is particularly evident in the case of Mirkat211 based sample, that reaches the highest conversion (70%) and for the P25/SBA-15 composite where EB conversion increased around for times.

The results indicate that the use of an ordered mesoporous silica as support effectively improved the catalytic activity, possibly due to an improved TiO_2_ dispersion, which might enhance the interaction among the catalyst and photons.

**Figure 5:**
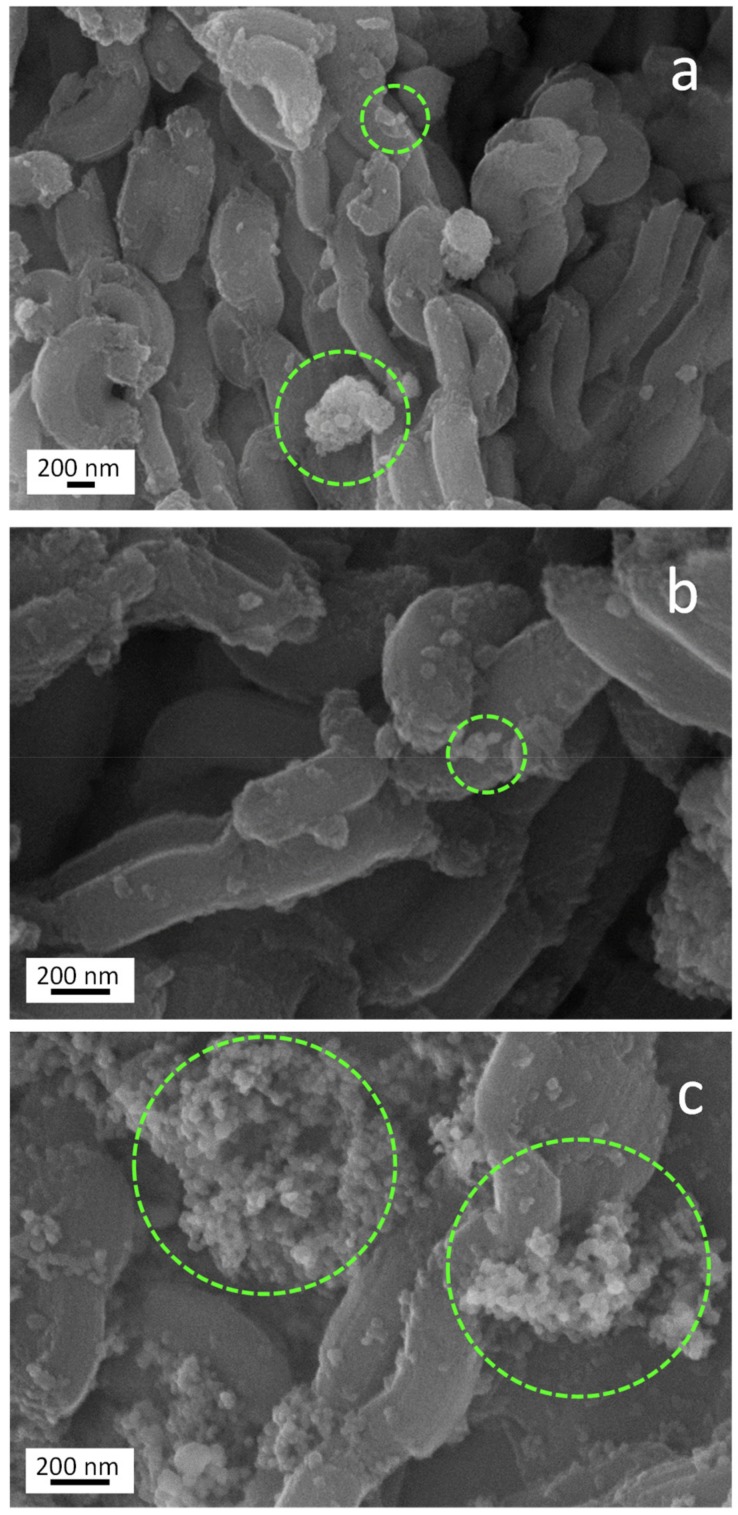
SEM images of Kronos VLP/SBA-15 (**a**) Mirkat 211/SBA-15, (**b**) and P25/SBA-15, and (**c**) materials.

In order to establish structure-activity relationships for further sustainable green building applications, the morphological, textural, and structural features along with the electronic properties and the hydrophilicity of the materials were deeply investigated. As shown in Figure S2, sections a, c and e, all the TiO_2_/SBA-15 samples exhibit quite similar morphology consisting of a long fibrous macrostructure, extending tens of micrometers, made of short clusters of rods with relatively uniform size. Such kind of morphology is generally known to be exhibited by materials having long-range mesostructure, typical of the siliceous support [34]. The presence of elongated crystals was observed (see sections b, d and f of the same Figure), confirming the strong influence of the silica matrix on the final morphology of the materials. Moreover, TiO_2_ nanoparticles can be clearly observed at higher magnification on the silica surface of each system (see Figure 5).

In particular, TiO_2_ nanoparticles (highlighted by green dashed circles) appear as bright particles of rounded shape with jagged edges. These nanoparticles seem to be better dispersed in the case of Mirkat 211- and Kronos VLP-containing samples (sections a and b). On the contrary, given the same TiO_2_ loading, titania nanoparticle agglomerates with bigger size were observed in the material synthesized with P25 (section c of the same Figure), indicating that P25 favors the constitution of a less dispersed material.

The beneficial effect of TiO_2_ on SBA-15 external surface only was supported by surface area and porosity data from physisorption analyses. The results are summarized in Table 2 and are compared to those obtained for the SBA-15 alone.

All materials exhibit an irreversible type IV isotherm (Figure S3, black curve) with a clear type-H2 hysteresis loop that is typical of materials with cylindrical mesopores. In addition, the isotherm displays a sharp inflection occurring at 0.7 < *p/p_0_* < 0.9, corresponding to the capillary condensation in the mesopores, which strongly suggests the presence of pores with size of about 8 nm. The sharpness of the hysteresis indicates a uniform pore size and a narrow distribution. Nonetheless, the composite photocatalysts isotherms (pink, blue, and green curves) did not change significantly compared to the bare SBA-15. Indeed, according to the data reported in Table 2, the values of surface area and porosity of the TiO_2_/SBA-15 samples were comparable to those obtained for SBA-15 of silica, indicating that the textural properties of the ordered mesoporous silica were preserved after impregnation. In particular, the values obtained for BET surface area, the pore size and volume of Mirkat 211/SBA-15 are the most similar to those of bare SBA-15.

The above evidences confirm that titania nanoparticles are located at the outer surface of the siliceous matrix and overall do not fill any pore (this is consistent with the results obtained with SEM images). It is worth noting that if the TiO_2_ nanoparticles were located within the channels of silica, the resulting material would not have been active, since titania would not be available to be photoactivated and thus to catalyze the degradation of ethylbenzene. Titania nanoparticles size was evaluated by Rietveld refinement and the results are reported in Table 2. The Mirkat 211/SBA-15 composite contains titania nanoparticles with smaller size than those of the two other samples, in agreement with the trends of the photocatalytic activity. Nevertheless, the differences observed for the textural and structural properties of the composite materials are too contained to justify the differences in the photocatalytic activity.

**Figure 6:**
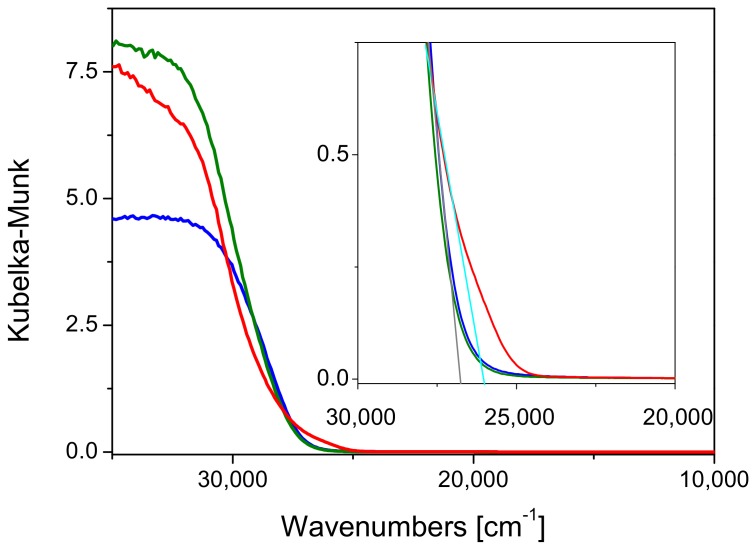
DRUV-Vis spectra of P25/SBA-15 (red line), Kronos VLP/SBA-15 (blue line) and Mirkat 211/SBA-15 (green line).

DRUV-Vis measurements were performed to check possible changes occurred on the electronic properties upon the insertion of titania on the SBA-15 support and the results are shown in Figure 6.

The band-gap values of all composite materials were larger than those estimated for the commercial titania samples, as shown in Figure 6, where the band-gap values of all samples are contrasted. The same observations were made in a recent paper [35] in which the position of the absorption edge was evaluated for TiO_2_ catalysts supported on SBA-15 in comparison with commercial TiO_2_ P25. Indeed, in this case, the band gap values were determined from the extrapolation of the slopes of the modified K-M functions versus energy to zero absorption (indirect allowed transition method, *n* = 2). However, the authors found that the TiO2/SBA-15 catalyst revealed an increase in the Eg (3.30 eV), which was ascribed to the smaller size of TiO2 particles in the prepared catalyst supported on SBA-15 [36,37]. In particular, in this study the variation of band gap (ΔEg) had not the same extent depending on the different titania precursors, but the following trend was observed: P25 > Kronos VLP > Mirkat 211. A similar trend was observed as for the photocatalytic activity improvement upon the insertion in SBA-15 (P25/SBA-15: +3.6%; Kronos VLP/SBA-15: +1.2% ~Mirkat 211/SBA-15: + 1.6%). These features can be taken as an indication that (i) the electronic properties of the different titania samples underwent to a change upon insertion into the SBA-15 support, and (ii) such effect seems to be more contained in the case of the Mirkat 211/SBA-15 material. It can be proposed that small titania nanoparticles (under the XRD detection limit) are embedded in a “hard electron” material as insulating silica, where these nanoparticles can exploit more efficiently their photocatalytic activity. The interaction between the two materials likely occurs through OH groups, as investigated and discussed in detail in the following.

Moreover, the synergy between titania and silica can lead to an optimal ratio between the hydrophilic and hydrophobic features of the material here formulated. The hydrophilic–hydrophobic balance of a material affects vapor transmission (that is related with the water vapor migration and condensation in buildings) [38]. Therefore, also this parameter must be taken in consideration during the formulation of an insulating material used with a structural function in a building or as paint or varnish additive. Finally, after having considered assessed the improvements of performances due to the increase of surface area and TiO_2_ crystallite sizes, surface hydrophilicity was considered. In order to remove water from the surface and to investigate the hydrophilicity of the materials, that is a crucial parameter for an insulating material, further FTIR experiments were performed on all the composite materials. The spectra were collected in air and outgassed at increasing temperature (80 °C, 100 °C, 120 °C, 150 °C, and 170 °C) up to 200 °C, starting from room temperature (spectra collected immediately and after 30′ outgassing) up to 200 °C, and keeping the temperature for 10′ at each step. The same experiments have also been carried out on the commercial samples as well as on bare SBA-15 for comparison.

Unexpectedly, the comparison among the series of FTIR spectra reported in Figure S4 and related to Mirkat 211 (red lines), SBA-15 (wine lines), and Mirkat 211/SBA-15 (orange lines) obtained upon outgassing the samples at increasing temperature reveals that the addition of titania to SBA-15 produced a modification in the spectrum profiles. Such modification can be ascribed to the change of the refraction index, as a consequence of the change of the size of the nanoparticles of the material, that is occurring at all the temperatures here considered, from r.t. (dashed bold lines, orange curves vs. red curves) up to 200 °C (bold lines, orange curves vs. red curves). The appearance of such feature may be possibly due to the effect of ultrasound irradiation to which the different commercial titania samples were submitted before impregnation of SBA-15. If compared to Kronos VLP/SBA-15 (less pronounced, Figure S5) and P25/SBA-15 (almost nihil, Figure S6), this phenomenon is mostly occurring in the case of Mirkat 211/SBA-15. This could be reasonably explained by the original small size and high surface area of the Mirkat material among the commercial titania samples and follows the order: Mirkat 211/SBA-15 > Kronos VLP/SBA-15 >> P25/SBA-15.

**Figure 7:**
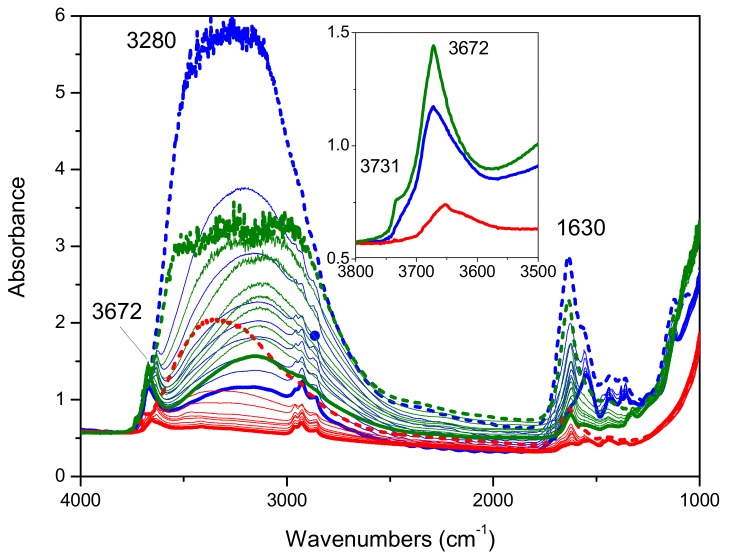
FTIR absorbance spectra collected on Mirkat 211 (green lines), Kronos VLP (blue lines) and P25 (red lines) in air (bold dashed lines) and upon outgassing at room temperature (immediately and after 30′) and at increasing temperature (80 °C, 100 °C, 120 °C, 150 °C, and 170 °C) up to 200 °C (bold lines). Spectra normalized on the density of the pellets.

In Figure 7 the FTIR spectra collected on Mirkat 211 (green lines), Kronos VLP (black lines) and P25 (blue lines) upon outgassing from room temperature up to 200 °C are shown. Upon increasing the temperature, a gradual decrease in intensity of the broad absorption at about 3280 cm^−1^ as well as of the peak at 1630 cm^−1^, due to the presence of water, is observed in all series of spectra collected on all the bare commercial titania samples.

At room temperature, the hydrophilicity of the samples follows the order: Kronos VLP > Mirkat 211 >>P25, whereas at high temperature (bold lines) the Mirkat 211 sample is able to retain more water molecules than the Kronos VLP sample. It also exposes the highest amount of free OH groups, according to the intensity of the peak at 3672 cm^−1^ (with a shoulder at 3731 cm^−1^).

**Figure 8:**
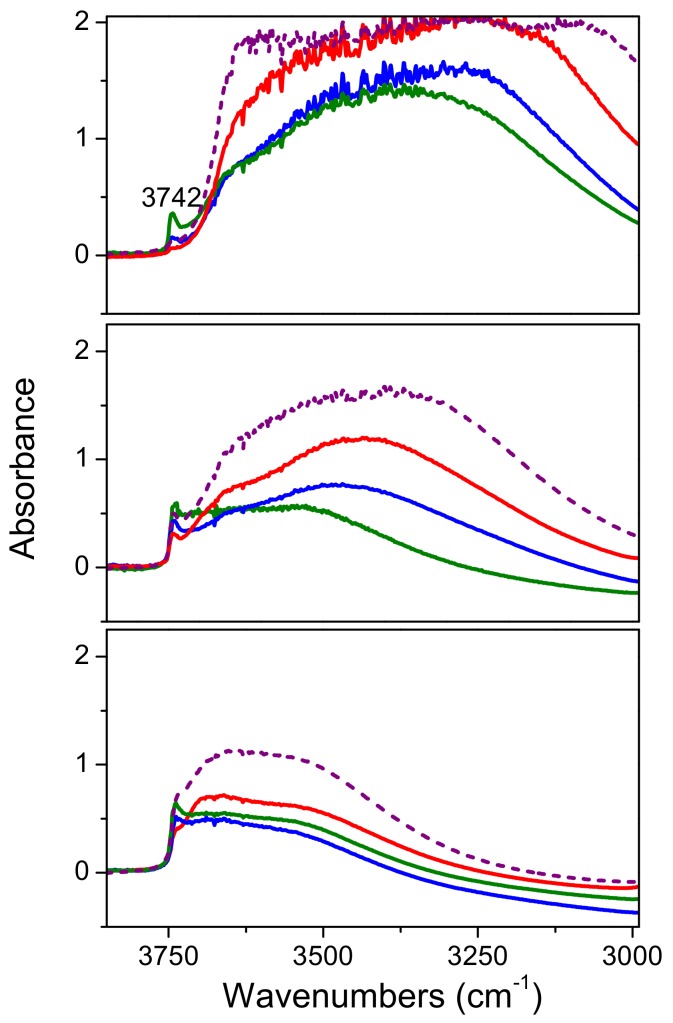
FTIR absorbance spectra of Mirkat 211/SBA-15 (green lines), Kronos VLP/SBA-15 (blue lines), P25/SBA-15 (red lines) and bare SBA-15 (dashed purple lines) collected in air (section a), upon outgassing at room temperature (section b) and at 200 °C (section c). Spectra normalized on the density of the pellets.

The same experiments performed on the composite samples (Figure 8) revealed that if compared to the bare commercial titania samples (Figures S4–S6) the hydrophilicity of these materials resulted enhanced by the presence of the SBA-15 matrix. Moreover, the free OH groups (peak at 3742 cm^−1^) observed are those related to the bare silica (see the comparison with the dashed red lines in sections a-c of Figure 7). However, upon outgassing at increasing temperature, the Mirkat 211/SBA-15 sample has the highest amount of free OH groups, but the lowest amount of OH groups in interaction with water molecules (broad absorption in the 3650–3000 cm^−1^ range).

These features indicate the occurrence of an interaction between the commercial titania samples and SBA-15, resulting in a different hydrophilicity of the composite materials. Such interaction seems more evident in the case of Mirkat 211/SBA-15, the material that benefited the most from deposition of titania on SBA-15 in ethylbenzene oxidation tests.

Finally, beside the improvement of the photocatalytic activity, the attainment of high surface area materials would be also advantageous in view of enhanced thermal insulation. Following this approach, the comparison among the FTIR transmittance spectra of the P25/SBA-15 (pink line), Kronos VLP/SBA-15 (blue line) and Mirkat 211/SBA-15 materials is shown in Figure S7. The spectra were normalized to the density of the pellets, therefore the differences observed as for the overall intensity of the spectra can be taken as a measure of the scattering capability of each material. The scattering is closely related to the size of the particles constituting the pellet: The larger the particles, the higher the scattering (given the same applied pressure to make the different pellets). These results are in agreement with the XRD and BET findings.

### 2.3. Insulating Features: Specific Heat Capacity

**Figure 9:**
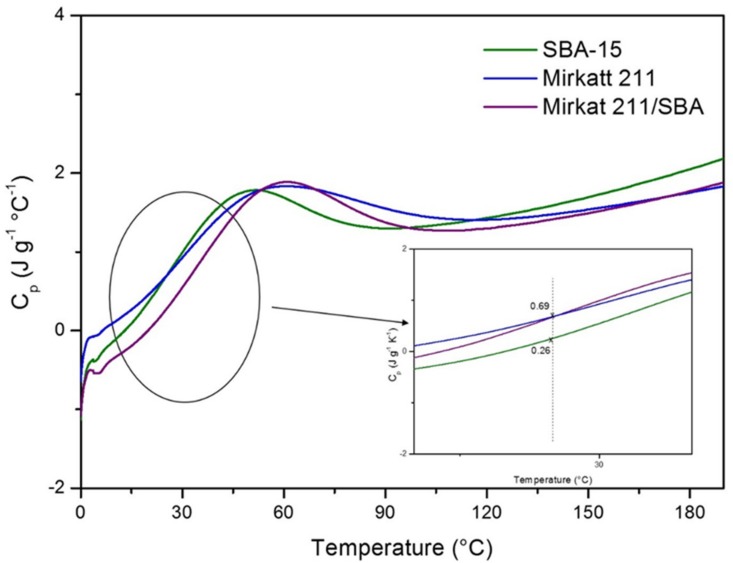
Heat capacity measured by modulated DSC (MDSC) for SBA-15, Mirkatt 211, Mirkat 211/SBA-15.

Thermal insulation is the reduction of heat transfer (i.e., the transfer of thermal energy between objects of differing temperature) between objects in thermal contact or in range of radiative influence. In the current context of continued growth in energy prices worldwide and concern to support global efforts to improve the climate, insulation of the exterior or interior walls of buildings is a well-known strategy to increase the energy efficiency of buildings. Thermal insulation can be achieved with specially engineered methods or processes, as well as with suitable object shapes and materials. As previously reported, one of the goals of this work is to formulate multifunctional materials able to improve the safety of building by the VOC abatement and by acting on insulation. The insulating capability of a material is measured as the inverse of thermal conductivity. Low thermal conductivity is equivalent to high insulating capability (Resistance value). In thermal engineering, other important properties of insulating materials are product density and specific heat capacity. This last parameter is defined as the amount of heat needed to raise the temperature of 1 kg of the material by 1 °C. A good insulator has a higher heat capacity because it takes time to absorb more heat before it actually heats up (temperature rising) to transfer the heat. In order to evaluate the potential insulating performance of the titania–silica composites we carried out a preliminary characterization by modulated DSC to investigate the heat capacity of pristine SBA-15, of commercial titania and of a titania–silica composite. The heat capacity (cp) of the materials as a function of temperature is reported in Figure 9.

The punctual heating capacity values at 25 °C are different for the three materials (see inset of Figure 8). SBA-15 exhibits the highest value (0.69 J g^−1^ K^−1^) that is in accord with experimental results reported in literature for other ordered mesoporous silica. Moreover, it is to note that this value is comparable with that of insulating materials used, traditionally, for building application (fiberglass 0.70 J g^−1^ K^−1^, concrete-cast dense 0.84 J g^−1^ K^−1^, granite concrete 0.82 J g^−1^ K^−1^). The silica–titania composite presents, instead, a heat capacity lower of pristine silica in all the range of investigated temperature. It must be considered that the calculation of the specific heat capacity value is closely related to the homogeneity and to the granulometry of the samples, but the experimental procedure used to press the materials (see experimental section) does not guarantee a good control of these features. The sample preparation was particularly hard in the case of the Mirkat 211/SBA15 catalyst.

To have a more reliable measure and comparison of the insulating features of the samples it must be considered the slope of the calorimetric curves. Silica–titania composite and SBA-15 profiles show the same trend with a marked slope, faster than that of Mirkat-211 calorimetric curve (this is highlighted in the Figure inset). This means that for these samples the temperature growth rate is higher. Consequently, both the materials require more energy to raise its temperature therefore having a higher heat capacity, showing a higher ability to resist the flow of energy through the material. Authors should discuss the results and how they can be interpreted in perspective of previous studies and of the working hypotheses. The findings and their implications should be discussed in the broadest context possible. Future research directions may also be highlighted.

## 3. Materials and Methods

### 3.1. Formulation and Preparation of the TiO_2_/SBA-15 Systems

Three TiO_2_ commercial photocatalysts, which differ in physicochemical properties, were used:

1) P25 Evonik (Degussa) is made by titanium dioxide in which two different crystalline phases with a 70/30 anatase/rutile ratio are present. In addition, recent studies demonstrated the co-presence of a small percentage of amorphous, that varies according to the production lot, usually <10%.

2) Mirkat 211 (Euro Support Manufacturing). This commercial sample is mainly composed by TiO_2_ (85%), but only 35%–40% is in the form of anatase phase, the rest is amorphous and contains a small percentage of SO_3_ that does not exceed 2 wt%.

3) Kronos VLP 7000 (Euro Support Manufacturing) is employed for the abatement of pollutants under visible light irradiation and it is active above 400 nm. This feature makes it one of the most performing photocatalysts on the market: It is prepared from titania with organic carbon added, followed by a thermal treatment.

In order to maximize porosity and surface area of the systems by improving at the same time photocatalytic activity and insulating features, each commercial photocatalyst was supported on a high surface area material that met the sustainability requirements. To this purpose, ordered mesoporous silica (SBA-15) was synthesized by using the procedure optimized in our laboratory and reported previously [38,39,40].

Briefly, SBA-15 was synthesized using tetraorthosilicate (TEOS) as silica precursors and EO20- PO70-EO20 (P123) (Aldrich) as a template, which was dissolved in aqueous HCl solution. The resultant mixture was treated hydrothermally at 90 °C for 48 h. The obtained solid was washed and then calcined at 550 °C for 6 h under air flow.

Therefore, starting from the commercial titania and the synthesized SBA-15, the TiO_2_–SiO_2_ systems were prepared by a quite simple incipient wetness impregnation procedure. The synthetic approach is low cost, sustainable and scalable and it satisfies the requirements for the green building.

**Figure 10:**
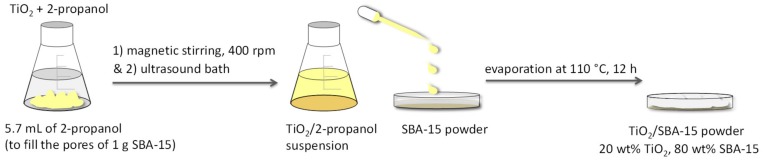
Main steps of the incipient wetness impregnation to obtain the TiO_2_/SBA-15 materials.

According to Figure 10, TiO_2_ was added to the suitable amount of 2-propanol, that was calculated on the basis of the volume of liquid necessary and sufficient to fill all the pores of the silica support (1 g of SBA-15: 5.7 mL of solvent).

The TiO_2_/2-propanol suspension was homogenized under magnetic stirring in a vial at 400 rpm and a further homogenization was performed by using an ultrasonic bath. Then, the obtained suspension was added dropwise to the SBA-15 powder until the total impregnation of the pores. The solvent was eliminated by evaporation at 110 °C for 12 h. The final amount of titania was 20 wt% for all photocatalysts.

### 3.2. Photocatalytic Tests on VOC Abatement

**Figure 11:**
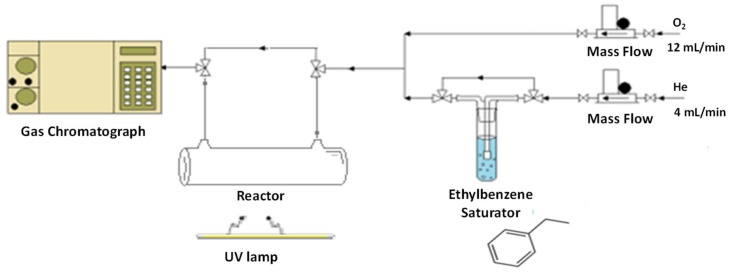
Scheme of the experimental apparatus.

The photocatalytic activity in gas phase VOC abatement was evaluated by considering the ethylbenzene (EB) molecule oxidation as model reaction. In particular, the photoactivity was evaluated using the experimental apparatus reported in Figure 11.

The reaction has been carried out by using a borate glass cylindrical thin film reactor (diameter 10 mm, length 100 mm). The catalyst (20 mg) was introduced by deposition of the catalyst suspension in 2-propanol on the light-exposed side of the reactor. The samples were irradiated by using a 125 W mercury UVA lamp (purchased from Helios Italquartz s.r.l. with emission range 315–400 shielded by a special tubular quartz, in order to select the 366 nm wavelength) and the measured irradiance on the sample was 2.4 mW/cm^2^. It has to be noted that by this approach the diffusion limitations typical of many photocatalytic tests are completely removed.

A typical experiment was performed at room temperature, with the following gas feed composition: 75% O2-25% He, in the presence of 1000 ppm EB, with a linear gas flow rate of 16 cm^3^/min and a sample mass of 20 mg. The gas analysis composition was performed by on-line gas-chromatograph equipped with a Porapak Q column and a TCD detector.

Blank tests were carried out in the absence of either light, catalyst, oxygen, and EB. CO_2_ evolution was observed in none of these cases, indicating that EB oxidation is a pure photocatalytic process and that the catalyst is photostable.

**Figure 12:**
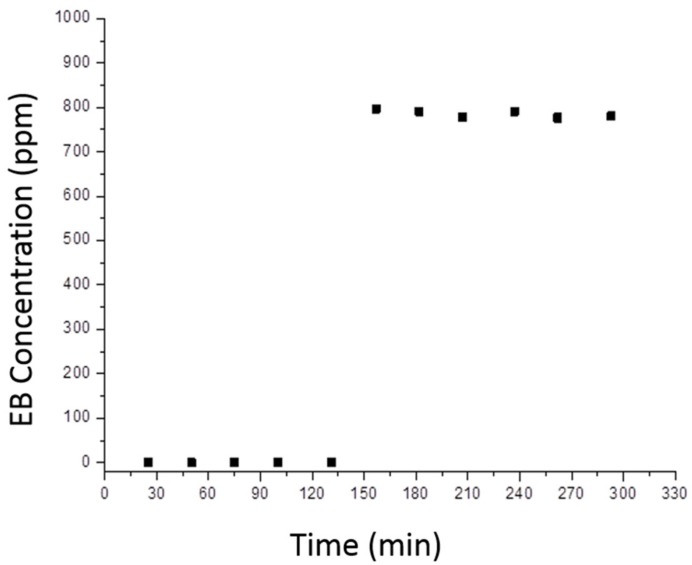
Typical model with the amount of non-reacted ethylbenzene as a function of time.

A characteristic reaction profile is shown in Figure 12, where two distinct reaction steps are clearly visible. In the former one the concentration of detected EB is zero, indicating that all molecules that reach the surface of the catalyst are absorbed: this process continues until the full coverage of the available superficial sites is reached and this evidence was observed also in the blank tests in the absence of irradiation. Moreover, the temporal extent of absorption is an indication of the total amount of absorbed organic molecules. Nonetheless, in this initial phase EB is not only adsorbed, but also partially converted to CO_2_ and H_2_O, however adsorption kinetics is predominant on the whole process. Indeed, the TiO_2_-containing photocatalyst progressively darkened, due to cracking of the organic compound under UV light irradiation.

In the latter reaction step, in which adsorption-desorption equilibrium has been reached, EB is detected and, in particular, the output amount of pollutant is lower than the input amount and the decomposition to CO_2_ and H_2_O is continuing. This happens after the total covering of the photocatalyst surface.

The ethylbenzene conversion (X_EB_) was calculated according to the following equation (Equation (1)):

(1) $$X_{\mathrm{EB}}=\frac{EB_{0}-EB\left(t\right)}{EB_{0}}$$

where EB_0_ is initial EB concentration while EB(t) is the concentration at the specific reaction time t. Conversion is measured when adsorption-desorption equilibrium is reached.

All the catalysts were tested at least twice in order to verify both reliability of the results and reproducibility of the synthetic procedure. The photocatalytic results have been compared to those obtained with the commercial reference material.

### 3.3. Characterisation

The crystal structure of the as-synthesized samples was characterized by powder XRD with an automated Philips Bragg–Brentano diffractometer equipped with a graphite monochromator. The long-fine focus Cu tube was operated at 40 kV and 25 mA. Spectra were recorded in the 2θ range 20–100° with a 0.03° step and 14 s counting time. The structures were refined with the program General Structure Analysis System (GSAS) [41]. The reflection shape was modeled with a pseudo-Voigt function; the FWHM was refined as a function of 2θ taking into account both Gaussian and Lorentzian broadening. The background was modeled with a 9-terms polynomial function. Cell parameters, scale factor, and the background polynomial function were free variables during the refinement. Parameters were added stepwise to the refinement in the following order: 2θ zero-shift, peak shape, peak asymmetry, atomic coordinates, and isotropic thermal factor. The intensity cut-off for the calculation of the profile step intensity was initially set at 1.0% of the peaks maxima and were lowered to 0.1% in the final stages of the refinements. Final convergence was assumed to be reached when the parameter shifts were <1% of their respectively estimated standard deviation. Estimated errors, provided by the Rietveld refinement program, are ± 0.0002 Å for the cell parameters and ± 0.002 Å for the selected interatomic distances [42].

SEM images were obtained using a Field Emission Gun Electron Scanning Microscopy LEO 1525 (ZEISS), after metallization with Chromium. Elemental composition was determined using a Bruker Quantax EDS. The images were acquired at the acceleration voltage of 15 kV by inlens detector.

N_2_ adsorption-desorption isotherms at −196 °C were performed using a MICROMERITICS ASAP 2000 analyzer in order to obtain information on surface area and pore volume. Prior to N2 physisorption experiments, all samples were outgassed at 200 °C for 2 h. Mesopore volume was measured as the adsorbed amount of N2 after capillary condensation. Surface area was calculated using the standard Brunauer, Emmett, and Teller (BET) method [43] and pore size distribution was elaborated using the BJH method applied to the isotherms desorption branch [44].

For diffuse reflectance UV–Vis-NIR analysis, powders were placed in a quartz cell. Diffuse reflectance UV–Vis-NIR spectra were run at r.t. on a Varian Cary 5000 spectrophotometer, working in the range of wavenumbers 50000–4000 cm^−1^. The spectra are reported in the Kubelka–Munk function [f(R_∞_) = (1 − R_∞_)^2^/2R_∞_; where R_∞_ represents the reflectance of an “infinitely thick” layer of the sample.

The surface hydrophilicity of the photocatalysts has been investigated by means of transmission FTIR spectroscopy. The FTIR spectra were taken on a Perkin Elmer 2000 spectrometer (equipped with a cryogenic MCT detector) by using an AABSPEC 2000 cell allowing to run in situ spectra in controlled atmosphere and temperature. The spectra were collected under outgassing from room temperature (r.t.) up to 200 °C. All spectra were normalized to the density of the pellets and, where specified, also to the titania amount of the material.

The heat capacity (cp) of the materials as a function of temperature was measured using a DSC Instrument (TA, model Q200, modulated) in the range of temperature from 0 to 220 °C. Before the measures, the samples were pressed by an ATR kit in order to obtain disks of 7 mm of diameter. The measurements were carried out as follows: firstly, the heat flow patterns of each material were acquired by means of a DSC dynamic scan (ramp 3.00 °C/min to 220.00 °C, modulation ± 0.48 °C every 60 s) in a preliminary scan, in which the weight of the sample was measured before and after the scan. A second heating scan was performed on the same samples by normalizing the DSC flux to the real weight of the sample after the preliminary heating.

The heat capacity was calculated according to the following equation (Equation (2)):

C_p = 1/m ∂H/∂T = 1/m (∂H/∂t)/(∂T/∂t) = 1/m ΔP/β(2)

where ∂H/∂t is the heat flow, m is the mass of the sample p, ΔP is the signal of the DSC (W) and β is the heating rate.

## 4. Conclusions

An effective, green and inexpensive method was reported for the formulation of multifunctional materials (made up of silica and titania) for applications in the sustainable green building sector with both insulating and indoor air purifier functions. From experimental evidences, it is clear how the formulation approach maximizes the efficacy of both components. The co-presence of titania and SBA-15 has a synergic effect, possibly due to the improved interaction between photocatalytically active titania and incident light, resulting in an enhancement of the photoactivity for all the considered catalysts. On one hand, the collaborative effect among TiO_2_ surface area, crystalline phase, nanoparticle dispersion and electronic properties allowed to obtain a high-performing material in air pollutants abatement. On the other hand, the high surface area, provided by SBA-15, suggests a very low thermal conductivity comparable to that of aerogels, which are known to be the best insulating material on the market. Moreover, being purely inorganic, these materials are fire and draught proof, differently from commonly-used materials in green building. Finally, the synthesized composite materials possess an optimal hydrophilic-hydrophobic balance resulting from the synergic interaction between commercial titania and SBA-15. This feature is of pivotal importance to deliver suitable insulation effect.

The proposed formulation approach is simple, low cost and therefore appealing for industry applications. Moreover, such materials are perfectly in line with the requirements of the sustainability in the building sector.

## Figures and Tables

**Table 1:** Textural properties of commercial titania samples.

Sample	Specific Surface Area (m^2^/g)	Pore Size Range (nm)	Pore Volume (ml/g)	Crystallite Size *^a^* (nm)
**KRONOS VLP**	269	4–10	0.30	15
**MIRKAT 211**	217	4–10	0.27	11
**P25**	52	4–80	0.13	40

^a^ evaluated by XRD.

**Table 2:** Textural properties of the TiO2/SBA-15 materials along with those of bare SBA-15.

Sample	Specific Surface Area (m^2^/g)	Pore Size Range (nm)	Pore Volume (ml/g)	Crystallite Size *^a^* (nm)
**SBA-15**	852 ± 2	8.5	1.09	-
**KRONOS VLP/SBA-15**	802 ± 3	8.2	0.99	22
**MIRKAT 211/SBA-15**	810 ± 4	8.0	1.06	10
**P25/SBA-15**	797 ± 2	8.0	0.99	30

^a^ evaluated by XRD.

## Supplementary Materials

The following are available online at https://www.mdpi.com/1420-3049/24/23/4226/s1, Figure S1. SEM images of Kronos VLP (section a), Mirkat 211 (section b) and P25 (section c) commercial samples. Instrumental magnification of all images is 1000×; Figure S2. SEM images of Kronos VLP/SBA-15 (sections a and b), Mirkat 211/SBA-15 (sections c and d) and P25/SBA-15 (section e and f) materials. Instrumental magnification of all images is 1000× and 10,000 × (left column and right column, respectively); Figure S3. N2 adsorption-desorption isotherms obtained for SBA-15 (black line), P25/SBA-15 (red line), Kronos VLP/SBA-15 (blue line) and Mirkat 211/SBA-15 (green line); Figure S4. Comparison among the FTIR absorbance spectra of to Mirkat 211 (red lines), SBA-15 (wine lines) and Mirkat 211/SBA-15 (orange lines) collected in air and outgassed at increasing temperature (80 °C, 100 °C, 120 °C, 150 °C and 170 °C) up to 200 °C (bold lines), starting from room temperature (spectra collected immediately, dashed bold curves, and after 30′ ougassing) up to 200 °C, and keeping the temperature for 10′ at each step. Spectra normalized to the density of the pellets and to the Mirkat 211 and SBA-15 contents; Figure S5. Comparison among the FTIR absorbance spectra of to Kronos VLP (black lines), SBA-15 (violet lines) and Kronos VLP/SBA-15 (grey lines) collected in air and outgassed at increasing temperature (80 °C, 100 °C, 120 °C, 150 °C and 170 °C) up to 200 °C (bold lines), starting from room temperature (spectra collected immediately, dashed bold curves, and after 30′ ougassing) up to 200 °C, and keeping the temperature for 10′ at each step. Spectra normalized to the density of the pellets and to the Kronos VLP and SBA-15 contents; Figure S6. Comparison among the FTIR absorbance spectra of to P25 (blue lines), SBA-15 (violet lines) and P25/SBA-15 (navy lines) collected in air and outgassed at increasing temperature (80 °C, 100 °C, 120 °C, 150 °C and 170 °C) up to 200 °C (bold lines), starting from room temperature (spectra collected immediately, dashed bold curves, and after 30′ ougassing) up to 200 °C, and keeping the temperature for 10′ at each step. Spectra normalized to the density of the pellets and to the P25 and SBA-15 contents; Figure S7. FTIR transmittance spectra of P25/SBA-15 (blue line), Kronos VLP/SBA-15 (black line) and Mirkat 211/SBA-15 (green line) materials after 30 min outgassing at room temperature. Spectra normalized to the density of the pellets; Table S1. Band gap value of the commercial titania samples and of the composite materials.
