# Supplementary material for: Multifunctional and Environmentally Friendly TiO2–SiO2 Mesoporous Materials for Sustainable Green Buildings
Source: Molecules. 2019 Nov 20;24(23):4226. doi: 10.3390/molecules24234226 (PMC6930548; doi:10.3390/molecules24234226)
Supplement: Supplementary file 1 [file molecules-24-04226-s001.pdf]

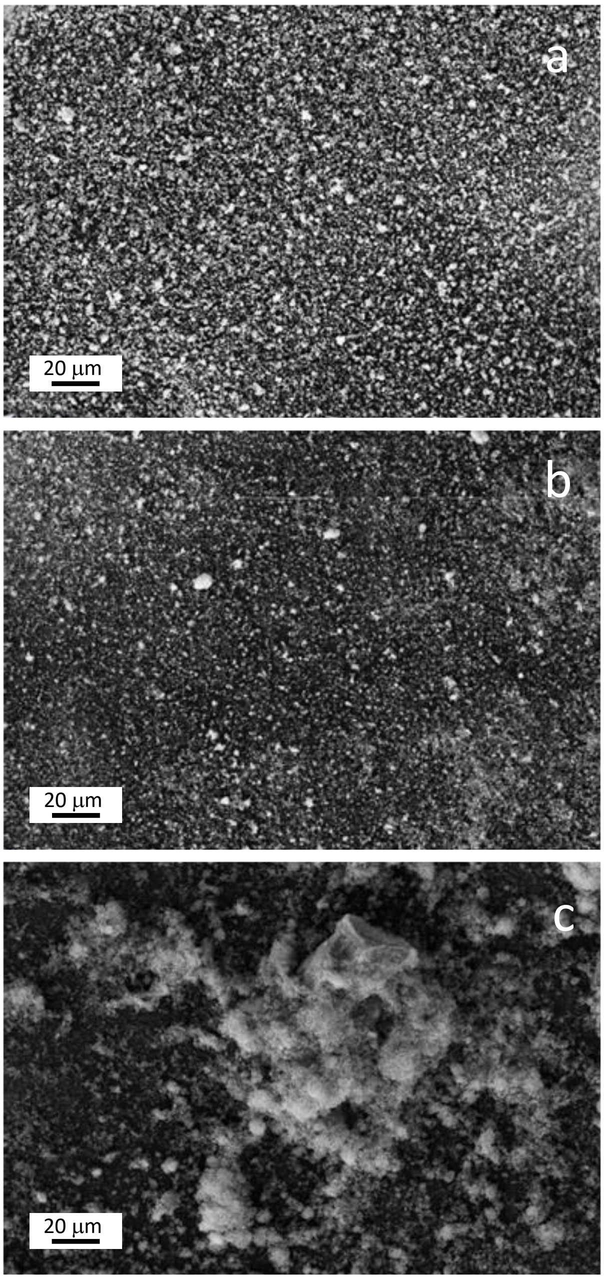

Figure SI-1. SEM images of Kronos VLP (section a), Mirkat 211 (section b) and P25 (section c) commercial samples. Instrumental magnification of all images is 1000×.

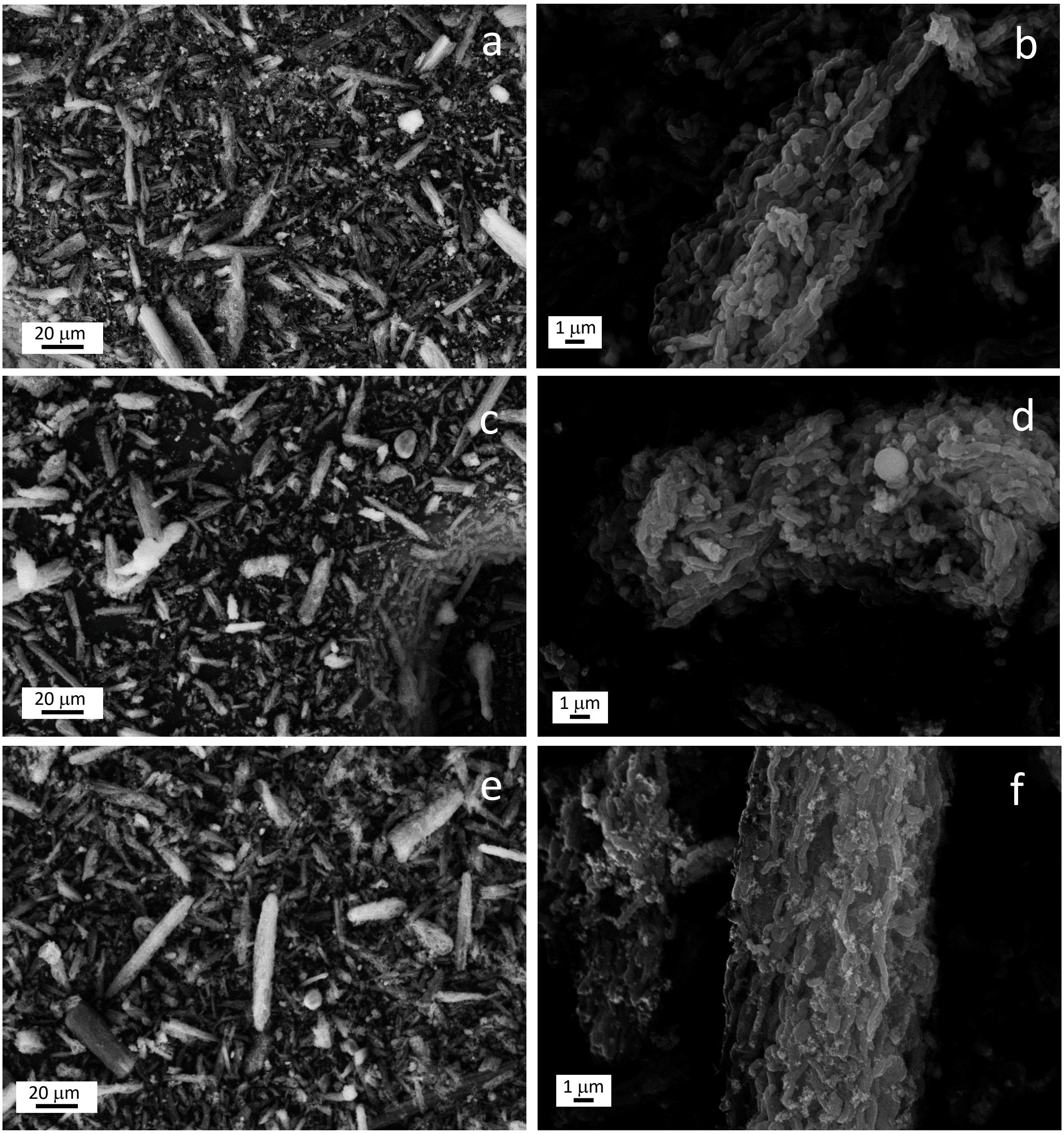

Figure SI-2. SEM images of Kronos VLP/SBA-15 (sections a and b), Mirkat 211/SBA-15 (sections c and d) and P25/SBA-15 (section e and f) materials. Instrumental magnification of all images is 1000× and 10,000 × (left column and right column, respectively).

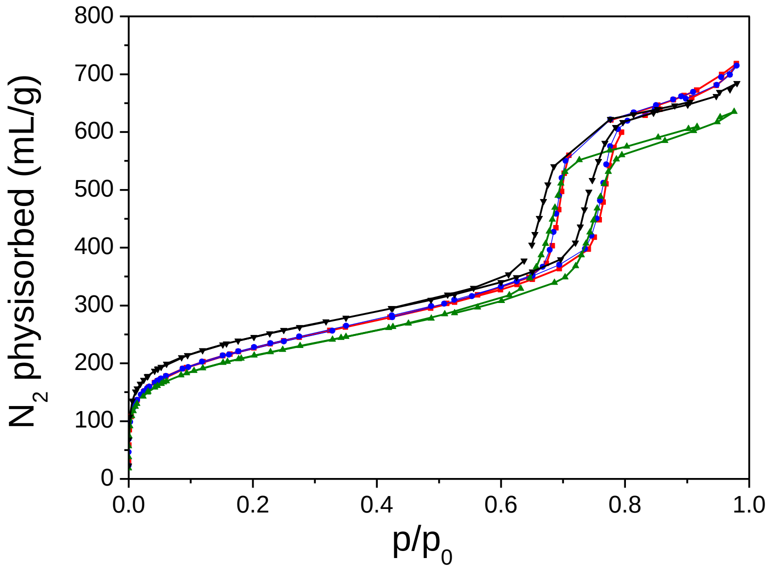

Figure SI-3. N_2 adsorption-desorption isotherms obtained for SBA-15 (black line), P25/SBA-15 (red line), Kronos VLP/SBA-15 (blue line) and Mirkat 211/SBA-15 (green line).

Table SI-1. Band gap value of the commercial titania samples and of the composite materials.

Commercial sample	Band-gap (eV)	Composite material	Band-gap (eV)	Δ (eV)
Kronos VLP	3.21	Kronos VLP/SBA-15	3.31	+0.10
Mirkat 211	3.25	Mirkat 211/SBA-15	3.32	+0.07
P25	3.15	P25/SBA-15	3.28	+0.13

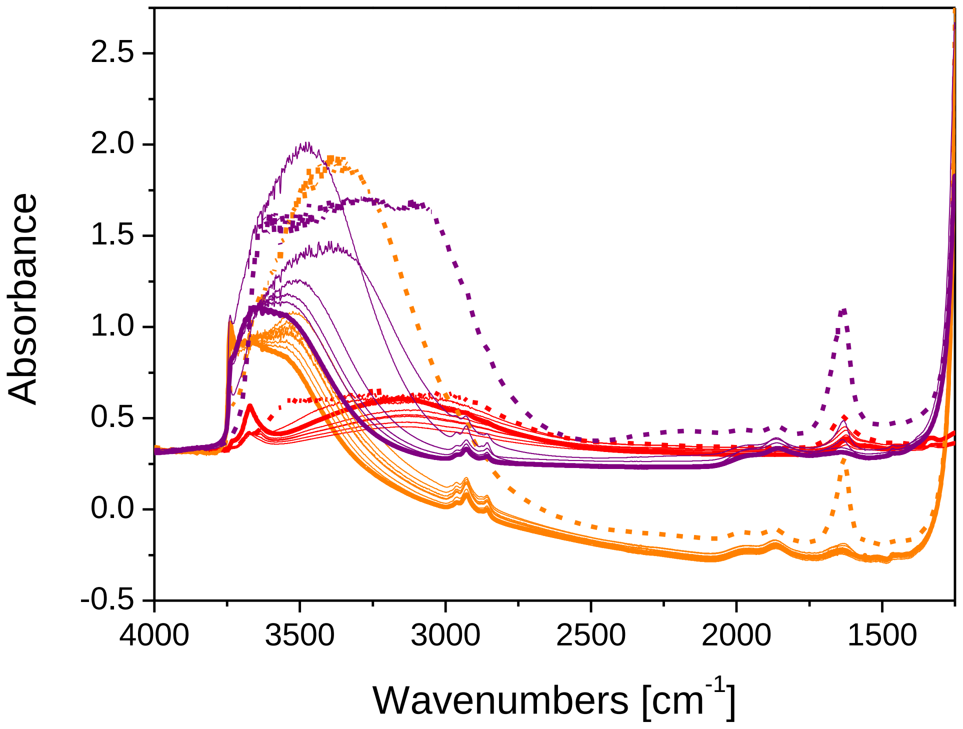

Figure SI-4. Comparison among the FTIR absorbance spectra of to Mirkat 211 (red lines), SBA-15 (wine lines) and Mirkat 211/SBA-15 (orange lines) collected in air and outgassed at increasing temperature (80°C, 100 °C, 120 °C, 150 °C and 170 °C) up to 200 °C (bold lines), starting from room temperature (spectra collected immediately, dashed bold curves, and after 30′ ougassing) up to 200°C, and keeping the temperature for 10′ at each step. Spectra normalised to the density of the pellets and to the Mirkat 211 and SBA-15 contents.

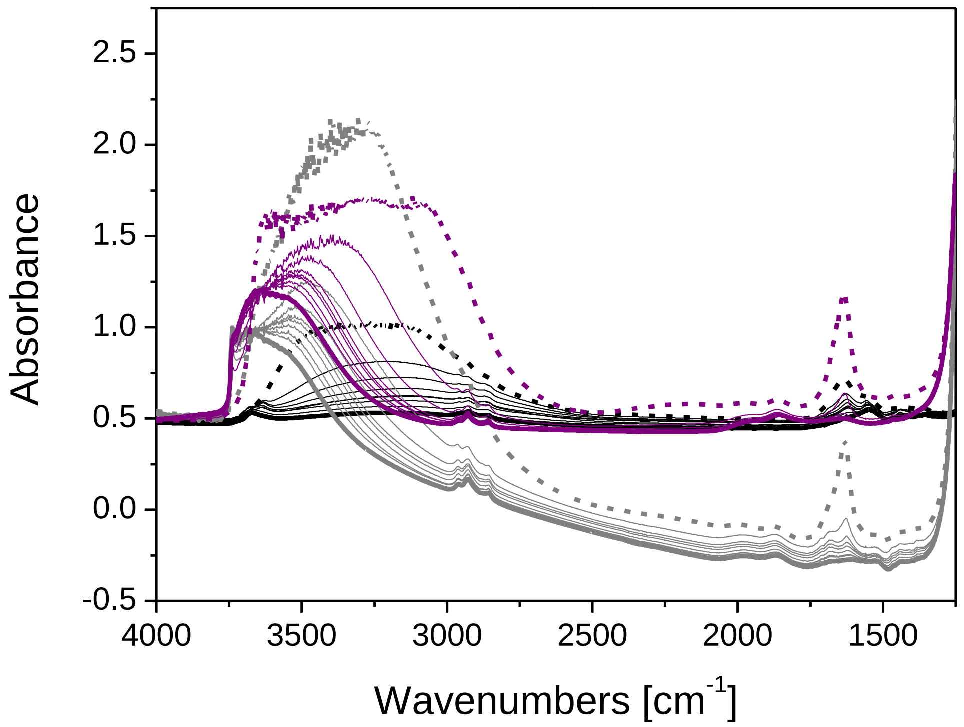

Figure SI-5. Comparison among the FTIR absorbance spectra of to Kronos VLP (black lines), SBA-15 (violet lines) and Kronos VLP/SBA-15 (grey lines) collected in air and outgassed at increasing temperature (80°C, 100 °C, 120 °C, 150 °C and 170 °C) up to 200 °C (bold lines), starting from room temperature (spectra collected immediately, dashed bold curves, and after 30′ ougassing) up to 200°C, and keeping the temperature for 10′ at each step. Spectra normalised to the density of the pellets and to the Kronos VLP and SBA-15 contents.

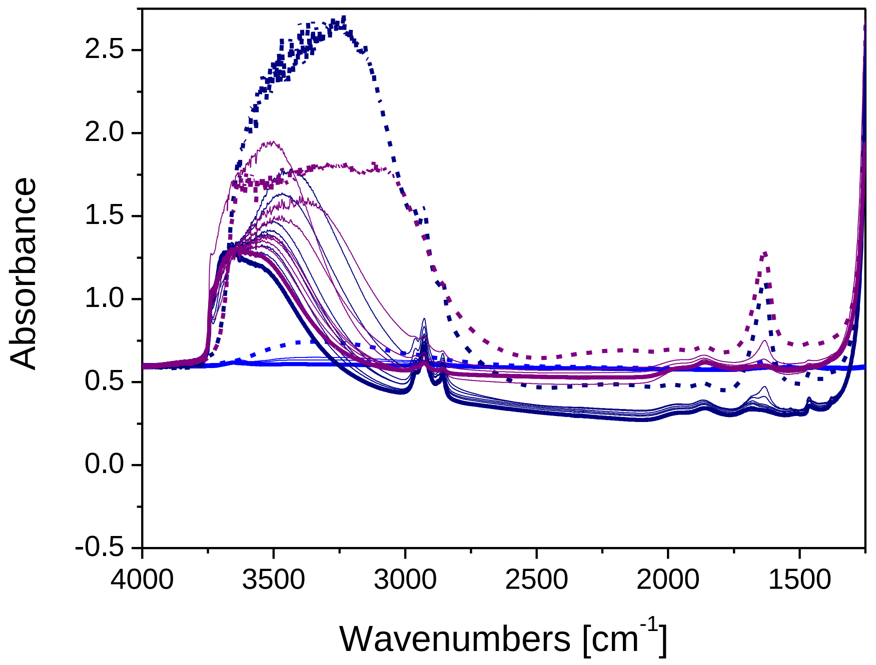

Figure SI-6. Comparison among the FTIR absorbance spectra of to P25 (blue lines), SBA-15 (violet lines) and P25/SBA-15 (navy lines) collected in air and outgassed at increasing temperature (80°C, 100 °C, 120 °C, 150 °C and 170 °C) up to 200 °C (bold lines), starting from room temperature (spectra collected immediately, dashed bold curves, and after 30′ ougassing) up to 200°C, and keeping the temperature for 10′ at each step. Spectra normalised to the density of the pellets and to the P25 and SBA-15 contents.

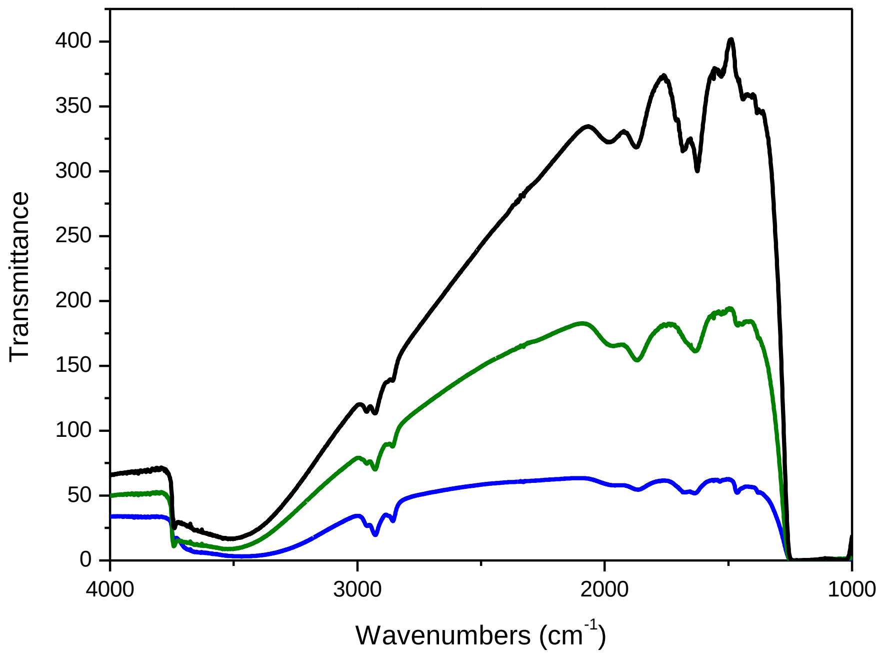

Figure SI-7. FTIR transmittance spectra of P25/SBA-15 (blue line), Kronos VLP/SBA-15 (black line) and Mirkat 211/SBA-15 (green line) materials after 30 minutes outgassing at room temperature. Spectra normalized to the density of the pellets.
